# Host–Gut Microbiome Metabolic Interactions in PFAS-Impacted Freshwater Turtles (*Emydura macquarii macquarii*)

**DOI:** 10.3390/metabo12080747

**Published:** 2022-08-16

**Authors:** David J. Beale, Thao V. Nguyen, Rohan M. Shah, Andrew Bissett, Akhikun Nahar, Matthew Smith, Viviana Gonzalez-Astudillo, Christoph Braun, Brenda Baddiley, Suzanne Vardy

**Affiliations:** 1 Land and Water, Commonwealth Scientific and Industrial Research Organisation, Ecosciences Precinct, Dutton Park, QLD 4102, Australia; 2 Oceans and Atmosphere, Commonwealth Scientific and Industrial Research Organisation, Battery Point, TAS 7004, Australia; 3 Land and Water, Commonwealth Scientific and Industrial Research Organisation, Research and Innovation Park, Black Mountain, ACT 2601, Australia; 4 NCMI, Commonwealth Scientific and Industrial Research Organisation, Battery Point, TAS 7004, Australia; 5 School of Veterinary Science, The University of Queensland, Gatton, QLD 4343, Australia; 6 Water Quality and Investigation, Science and Technology Division, Department of Environment and Science, Queensland Government, Dutton Park, QLD 4102, Australia

**Keywords:** freshwater turtles, *Emydura macquarii*, PFAS, water pollutants, omics, metabolomics, microbiome

## Abstract

Per-and polyfluoroalkyl substances (PFAS) are a growing concern for humans, wildlife, and more broadly, ecosystem health. Previously, we characterised the microbial and biochemical impact of elevated PFAS on the gut microbiome of freshwater turtles (*Emydura macquarii macquarii*) within a contaminated catchment in Queensland, Australia. However, the understanding of PFAS impacts on this species and other aquatic organisms is still very limited, especially at the host–gut microbiome molecular interaction level. To this end, the present study aimed to apply these leading-edge omics technologies within an integrated framework that provides biological insight into the host turtle–turtle gut microbiome interactions of PFAS-impacted wild-caught freshwater turtles. For this purpose, faecal samples from PFAS-impacted turtles (*n* = 5) and suitable PFAS-free reference turtles (*n* = 5) were collected and analysed. Data from 16S rRNA gene amplicon sequencing and metabolomic profiling of the turtle faeces were integrated using MetOrigin to assign host, microbiome, and co-metabolism activities. Significant variation in microbial composition was observed between the two turtle groups. The PFAS-impacted turtles showed a higher relative abundance of Firmicutes and a lower relative abundance of Bacteroidota than the reference turtles. The faecal metabolome showed several metabolites and pathways significantly affected by PFAS exposure. Turtles exposed to PFAS displayed altered amino acid and butanoate metabolisms, as well as altered purine and pyrimidine metabolism. It is predicted from this study that PFAS-impacted both the metabolism of the host turtle and its gut microbiota which in turn has the potential to influence the host’s physiology and health.

## 1. Introduction

*Emydura macquarii* is a species of freshwater short-necked turtle that primarily occurs in the Macquarie River basin and its associated waterways, extending to many coastal rivers of Queensland and New South Wales throughout central and eastern Australia [1,2]. Hence, it is often known as the Macquarie turtle or Murray turtle [3]. However, since the 1970s, populations have been in decline [4,5]. For example, in Australia’s southern Murray-Darling Basin alone, these short-necked turtles have declined by approximately 69% [4]. This decline could be attributed to nest predation by invasive foxes (*Vulpes vulpes*), environmental change/degradation, water management practices, and impacts of water pollutants [4,5,6]. As long-lived aquatic organisms and omnivores, turtles have the potential to bioaccumulate high contaminant loads from surrounding water and food sources in the river systems they inhabit, which may, in turn, lead to morbidity (i.e., reproductive consequences) and mortality [7,8,9,10]. Despite this, research into the effects of water pollutants, such as per- and polyfluoroalkyl substances (PFAS), on freshwater turtles and other reptiles and amphibians remain limited, especially at the molecular level.

PFAS are a large class of synthetic organofluorine chemicals that have been used in a wide range of consumer products and industrial applications since the 1940s [11,12]. They are highly persistent and prevalent in aquatic environments [13], and are known to bioaccumulate and cause harm to human and environmental health [12]. Therefore, it is no surprise that PFAS bioaccumulation has been reported in a variety of marine and freshwater aquatic organisms [13,14,15,16,17]. For example, elevated PFAS levels have been observed in different turtle species around the world [17,18,19,20,21,22]. As previously reported by this research group, perfluorooctane sulfonate (PFOS) bioaccumulation in turtle serum (*E. m. macquarii*) was measured at 889 ± 56 ng/mL, which was 235 times higher than water PFOS concentrations that the turtles were sampled from [22]. Morikawa et al. [21] reported a PFOS range of 2.4 to 486 ng/mL in wild-caught freshwater Red-eared Slider turtles (*Trachemys scripta elegans*) and Chinese Pond turtles (*Chinemys reevesii*) within a Japanese river system. De Solla et al. [18] reported alarmingly high PFAS levels of 2356 ng/mL in Snapping turtle (*Chelydra serpentina*) plasma near an international airport. However, the health of the PFAS-exposed turtles in the studies above was not assessed biochemically as intended herein.

Although the impact of PFAS on metabolic processes has been described in humans [23,24,25] and other animal models, such as water fleas (*Daphnia*) and zebrafish (*Danio rerio*) [26,27,28,29], this biological understanding remains limited for turtles (and other reptiles and amphibians). To date, a growing body of work by Beale and co-authors [22,30] describes alterations in the metabolic profiling of serum and oviducal eggs from turtles exposed to elevated PFAS, suggesting an inflammation response, metabolic preservation, and re-routing of central carbon metabolites. More recently, this also included a short communication paper on the faecal microbial compositions of PFAS-impacted turtles using 16S rRNA amplicon sequencing and metabolic profiling techniques [31]. In this study, indications of stress were observed; an elevated Firmicutes-to-Bacteroidetes ratio and metabolites tied to inflammation. However, insight into the host–gut microbiome connectivity and co-metabolic activities were not investigated. One avenue to further explore these relationships is via online integration tools such as MetOrigin [32]. MetOrigin facilitates the compartmentalisation of host, gut-microbiome, and co-metabolism activities tied to curated genome, sequenced microbiomes, and metabolomics databases (i.e., KEGG, HMDB, FoodDB, T3DB, etc.) [32].

To this end, the present study aims to build upon the descriptive characterization of the faecal microbiome presented in our previous short communication paper [31], to further apply an integrated multi-omics approach that deepens our biological understanding of the toxicological effects of PFAS on the host–gut microbiome metabolic interaction within wild-caught freshwater turtles. Liquid chromatography-mass spectrometry (LC–MS) based metabolomics approaches were used to analyse targeted central carbon metabolism metabolites and untargeted metabolites that were combined with 16S rRNA gene amplicon sequencing via MetOrigin to profile the host–gut microbiome function. The findings from this multi-omics study could provide useful information for future research that is targeted toward developing preservation strategies for *E. m. macquarii* in Eastern Australia.

## 2. Results

### 2.1. Turtle Physiology and Biochemistry

The average weight and carapace length of the sampled turtles from the impacted and reference sites were not statistically different. The average weight (±SD) and carapace length (±SD) for all sampled turtles were 1788 ± 217 g (*n* = 10) and 25.3 ± 1.0 cm (*n* = 10), with a student *t*-test *p*-value of 0.211 and 0.321 for weight and carapace length between sites, respectively. This is consistent with the size and weight of adult freshwater turtles for this species. All turtles were considered clinically healthy upon veterinarian physical examination; see Supplementary Photos. Turtle blood serum PFAS concentrations for the impacted turtles were Σ47 PFAS 1992 ± 620 ng/mL; reference turtles were below the limits of reporting [31].

A summary of the biochemistry outputs provided by a veterinarian clinical pathology laboratory (Reptile Biochemistry Comprehensive Panel Assay) is provided in Supplementary Table S1. The biochemistry panel assessment was considered within normal ‘wildlife’ ranges, with the caveat that this species has not been validated for the ‘Reptile Biochemistry Comprehensive’ assessment. Of note, glucose was considered low for the impacted and reference site turtles with 2.4 ± 0.9 (SD) mmol/L and 1.1 ± 0.8 (SD) mmol/L, respectively. This was likely due to a serum storage artifact and was not considered abnormal. Uric acid elevation was observed (*p*-Value > 0.001) in the impacted turtles (124 ± 5 µmol/L) and for reptiles is typically associated with mild dehydration. A significant difference in Glutamate Dehydrogenase (GLDH) (1.8 ± 0.8 (SD) IU/L; *p*-value = 0.041) was also observed and could be attributed to elevated uric acid and urea in the impacted turtles. Significant elevation (*p*-Value > 0.001) of Bile Acids Preprandial/Random in the impacted turtles were observed at 4.0 ± 0.7 (SD) µmol/L compared to 1.4 ± 0.5 (SD) µmol/L at the reference site. Again, all reported biochemistry was within ‘normal’ ranges.

### 2.2. Community Metabolomics Profile

**Figure 1:**
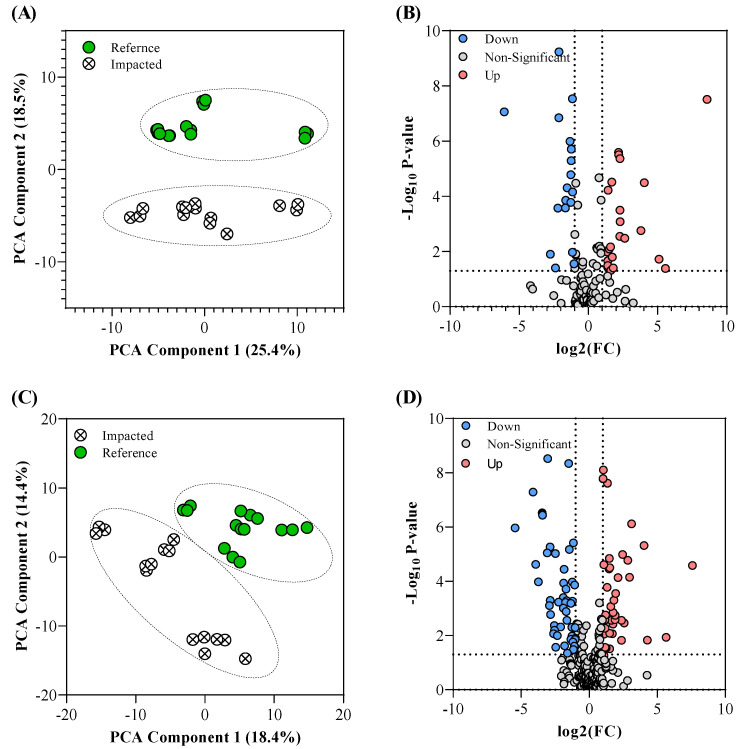
Multivariate comparison of the acquired metabolomic profile data from the reference and PFAS-impacted turtle faecal samples. Panels (**A**,**C**) are the Principal Component Analysis (PCA) of the turtle faecal microbiome samples using targeted Central Carbon Metabolism (CCM) metabolites and untargeted polar metabolites, respectively, from the reference site (green circles) and PFAS-impacted site (white crossed-out circles); Panels (**B**,**D**) are the volcano plots of the targeted CCM metabolites and untargeted polar metabolites data, respectively, identifying significant metabolites that are downregulated (blue circles) and upregulated (red circles) in the PFAS-impacted faecal samples with respect to the reference faecal samples. Note, a metabolite is considered significant if it is above the defined fold change (FC) threshold of 2.0 and below the *p*-Value cutoff of 0.05.

In total, 130 targeted central carbon metabolism (CCM) metabolites and 391 polar untargeted metabolites were measured across the sampled faeces. Figure 1A,C illustrate the principal component analysis (PCA) of these data, with Figure 1B,D providing an overview of the significant metabolite features (Fold change > 2.0, *p*-Value < 0.05).

**Figure 2:**
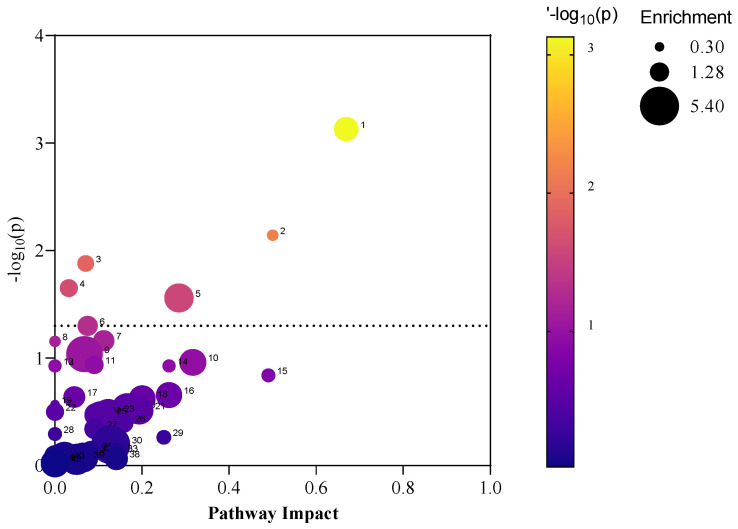
Pathway impact analysis using the over-representation enrichment data of the statistically significant metabolites from PFAS-impacted turtle faecal samples. Noting, (1) Alanine, aspartate and glutamate metabolism; (2) D-Glutamine and D-glutamate metabolism; (3) Arginine biosynthesis; (4) Butanoate metabolism; (5) Pyrimidine metabolism; and (6) Pantothenate and CoA biosynthesis. Note, Supplementary Table S4 provides names for the pathway features annotated 6–42.

Of the identified metabolites, 56 metabolites were upregulated in the impacted site samples compared to the reference samples, and 55 metabolites were downregulated in the impacted site samples compared to the reference samples. Supplementary Table S2 provides a summary of the significant metabolites identified by the volcano plot analyses. Where a metabolite was identified in both the targeted and untargeted approaches, the targeted data were selected (removing the untargeted data) before downstream processing. The significant metabolites were then used in an over-representation enrichment and pathway impact analysis. Figure 2 illustrates the pathway impact analysis output; Supplementary Tables S3 and S4 provide the enrichment and pathway analysis data that support Figure 2.

### 2.3. Microbiome Community Profile

**Figure 3:**
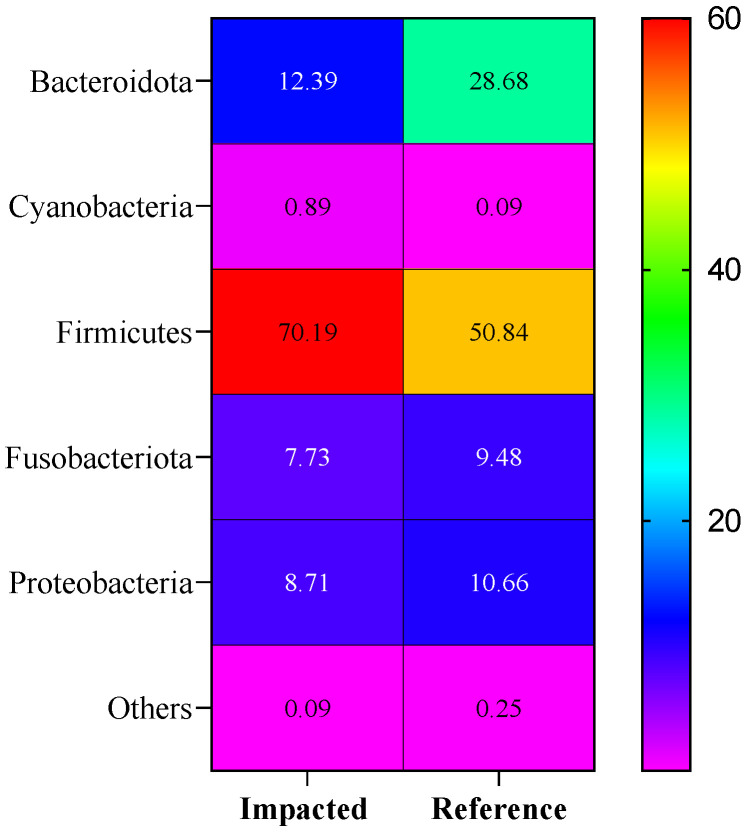
Relative bacterial phylum abundance in sampled freshwater turtle faeces per PFAS-impacted (*n* = 5 turtles) and reference (*n* = 5 turtles) site. Supplementary Table S5 provides the data to support this figure. Scale is relative abundance reported as a percentage (%).

The number of quality-filtered sequences obtained for each sample was 88,510–98,289, for a total of 945,308 sequences (the average number of reads per sample was 94,530). Data were filtered with a minimum median operational taxonomic units (OTU) abundance threshold of 4 reads, and a variance threshold of 10% based on the inter-quartile range. All rarefaction curves reached saturation (Supplementary Figure S1), indicating a sufficient sampling depth was achieved to cover the community diversity for each of the samples. Figure 3 provides an overview of the microbial community’s relative abundance at the phylum level.

### 2.4. Metabolite Origin Analysis

**Figure 4:**
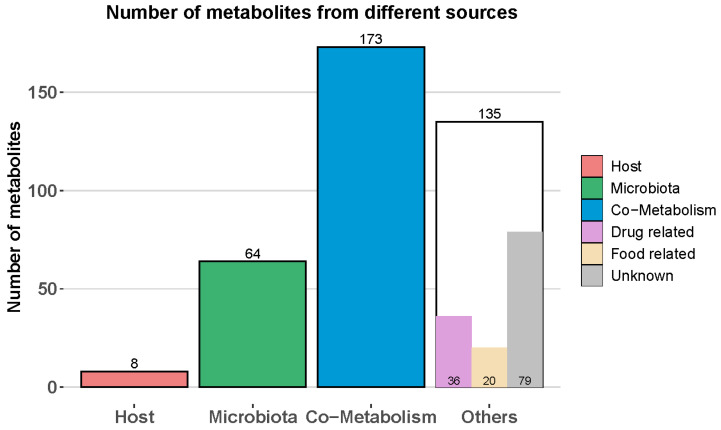
Number of identified metabolites from different sources as determined by MetOrigin (2.0) performed against the *Chrysemys picta* (Western Painted turtle) genome.

A metabolite host–microbiome origin analysis was performed against the *Chrysemys picta* (Western Painted turtle) genome within MetOrigin (v2.0). As illustrated in Figure 4, eight host-based metabolites, 64 microbiota-based, and 173 co-metabolism metabolites were assigned. An additional 135 metabolites related to food (36), drugs (20), and unknown origin (79) were also identified. The *Chrysemys picta* (Western painted turtle) genome was selected to perform the metabolite host–microbiome origin analysis as it is the nearest match to the turtle species under study herein.

Supplementary Table S5 lists the metabolite assignments per source and the chemical cluster ontologies as defined by ChemRICH, which correlate to androstanol (*n* = 2) and hydroxycholesterol (*n* = 2) originating from the host; dicarboxylic acids (*n* = 3), hydroxybenzoates (*n* = 3), phenols (*n* = 4), and sugar acids (*n* = 3) originating from the microbiome; and, butyrates (*n* = 6), disaccharides (*n* = 5), pyrimidine nucleosides (*n* = 5), and sugar acids (*n* = 4) originating from co-metabolic activities of the host and microbiome.

### 2.5. Host-Microbiome Function Analysis

**Figure 5:**
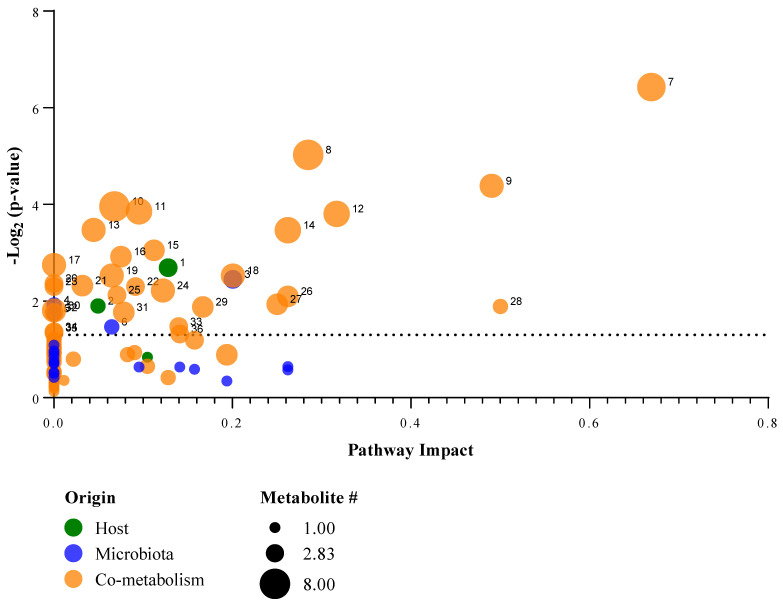
Pathway impact plot of the host–microbiome metabolic function metabolites. Noting, (1) steroid hormone biosynthesis; (2) tryptophan metabolism; (3) primary bile acid biosynthesis; (4) cysteine and methionine metabolism; (5) sulfur metabolism; (6) toluene degradation; (7) tyrosine metabolism (microbiota); (8) alanine, aspartate and glutamate metabolism; (9) pyrimidine metabolism; (10) vitamin B6 metabolism; (11) purine metabolism; (12) galactose metabolism; (13) glycine, serine and threonine metabolism; (14) pentose phosphate pathway; (15) glyoxylate and dicarboxylate metabolism; (16) beta-alanine metabolism; (17) pantothenate and CoA biosynthesis; (18) nicotinate and nicotinamide metabolism; (19) cysteine and methionine metabolism; (20) tyrosine metabolism (co-metabolism); (21) taurine and hypotaurine metabolism; (22) butanoate metabolism; (23) citrate cycle (TCA cycle); (24) carbon fixation in photosynthetic organisms; (25) arginine and proline metabolism; (26) arginine biosynthesis; (27) phenylalanine metabolism; (28) ascorbate and aldarate metabolism; (29) D-glutamine and D-glutamate metabolism; (30) aminoacyl-tRNA biosynthesis; (31) methane metabolism; (32) pentose and glucuronate interconversions; (33) glycolysis/gluconeogenesis; (34) terpenoid backbone biosynthesis; (35) carbon fixation pathways in prokaryotes; (36) inositol phosphate metabolism; and (37) lysine degradation.

The host–microbiome metabolic function analysis identified 37 significant metabolic pathways annotated for the host, microbiome, and co-metabolic activities (host and Microbiome) (Figure 5). Among these pathways of interest, there are 2 pathways from the host and 4 microbiome-based pathways, while the majority of these activities are co-metabolism-based (31). Amino acid metabolisms reflect the largest proportion of these pathways and tyrosine metabolism from microbiota is the most impacted activity. Supplementary Table S7 and Figure S2 provide all the identified pathways from the host–microbiome function analysis.

### 2.6. Metabolite–Microbiome–Host Correlation Analysis

**Figure 6:**
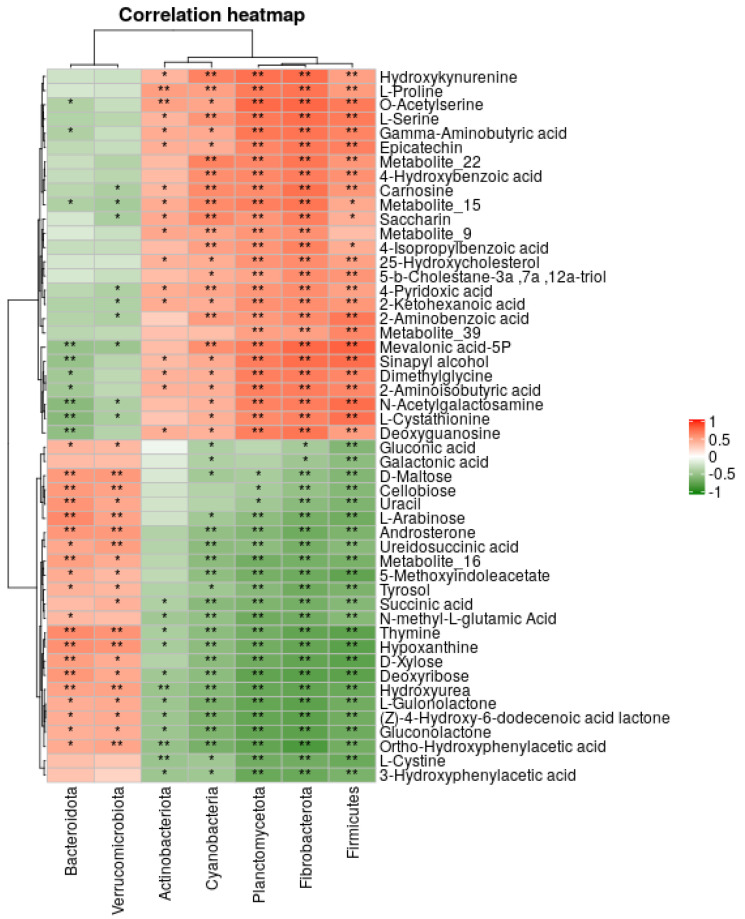
Microbiome community (at the phylum level) and expressed metabolite correlation analysis. Note, single asterisk (*) denotes a significance level of <0.05; a double asterisk (**) denotes a significance level of <0.01. Supplementary Figure S3 illustrates the correlation analysis at the species level.

The possible correlations between microbes and the identified metabolites were assessed via the Spearman rank correlation test. The microbes were clustered into two major groups that have opposite correlation directions with metabolites. While some metabolites were upregulated in one microbial group, they were downregulated in another group, and vice versa. At the phylum level (Figure 6), Bacteroidota and Verrucomicrobiota showed a similar correlation pattern with metabolites which was opposite to other phyla (Firmicutes, Fibrobacterota, Planctomycetota, Cyanobacteria, and Actinobacteriota). A similar correlation pattern was observed at the species level (Supplementary Figure S3). Six microbial species (*Bacteroidetes bacterium*, *Succinispira mobilis*, uncultured *Clostridum*, uncultured prokarypte, metgagenome, and uncultured bacterium) formed one group that had opposite metabolite correlation with the rest of the species analysed.

### 2.7. Metabolic Pathway Mapping

**Figure 7:**
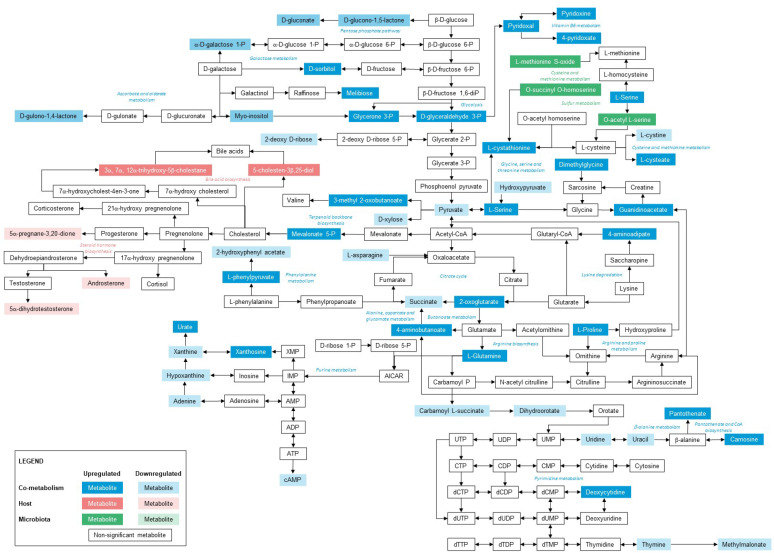
Pathway map highlighting the key pathways annotated to the host, microbiota, and co-metabolic activities of the PFAS-impacted turtles in relation to the reference turtle samples. Noting, the list of all significant metabolic pathways, metabolites involved and their KEGG IDs are enlisted in Supplementary Table S7.

There were 380 differential metabolites associated with PFAS exposure, including 8 host-specific metabolites, 64 microbiota-specific, 173 microbiota–host cometabolites, and 135 others (drug-related, food-related, and unknown) (Figure 4). Supplementary Table S7 provides all the identified pathways from the host–microbiome function analysis. Metabolic pathway enrichment analysis indicated 3, 20, and 76 related metabolic pathways that were matched against the host, microbiota, and co-metabolism pathway database (Supplementary Table S7); among which, 2, 4, and 30 metabolic pathways were identified as significantly (*p*-value < 0.05) associated with PFAS exposure. Based on metabolite origin-based analysis, steroid hormone biosynthesis and primary bile acid biosynthesis were specific to the host. The cysteine and methionine metabolism, sulfur metabolism, toluene degradation, and tyrosine metabolism were specific to the microbiota, and 30 metabolic pathways associated with carbohydrates, energy, amino acids, nucleotide, cofactors and vitamins, and terpenoids and polyketides were shared by both host and microbiota. Figure 7 illustrates the pathway map highlighting the significant metabolic pathways annotated for the host, microbiota, and co-metabolic activities. Supplementary Figures S4–S6 illustrates the host, microbiome, and co-metabolism networks and the feature abundances.

It was found that the top altered co-metabolic pathways were involved in alanine, aspartate and glutamate metabolism, pyrimidine metabolism, vitamin B6 metabolism, purine metabolism, galactose metabolism, glycine, serine and threonine metabolism, pentose phosphate pathway, glyoxylate and dicarboxylate metabolism, beta-alanine metabolism, pantothenate and CoA biosynthesis, nicotinate and nicotinamide metabolism, cysteine and methionine metabolism, tyrosine metabolism, taurine and hypotaurine metabolism, butanoate metabolism and citrate cycle (TCA cycle). The alanine, aspartate, and glutamate metabolism were altered via pyruvic acid (FC = 0.69), succinic acid (FC = 0.44), L-asparagine (FC = 0.1), oxoglutaric acid (FC = 3.41), L-glutamine (FC = 2.4) and GABA (FC = 2.34). Most metabolites in the pyrimidine metabolism decreased (uracil, FC = 0.51; thymine, FC = 0.31; uridine, FC = 0.78; dihydroorotate, FC = 0.67; carbamoyl L-succinate, FC = 0.31; methylmalonic acid, FC = 0.44) except L-glutamine (FC = 2.4) and deoxycytidine (FC = 1.87). Vitamin B6 metabolism was found to be upregulated via L-glutamine (FC = 2.4), glyceraldehyde 3-phosphate (FC = 1.74), pyridoxal (FC = 2.41), pyridoxamine (FC = 2.06) and 4-pyridoxic acid (FC = 3.25). The purine metabolism was altered via L-glutamine (FC = 2.4), adenine (FC = 0.78), hypoxanthine (FC = 0.31), deoxyguanosine (FC = 2.81), uric acid (FC = 20.94), xanthine (FC = 0.53), cyclic AMP (FC = 0.24) and xanthosine (FC = 1.23). Dihydroxyacetone phosphate (FC = 2.03), glyceraldehyde 3-phosphate (FC = 1.75), myo-Inositol (FC = 0.72), galactose 1-phosphate (FC = 0.99), sorbitol (FC = 5.94) and melibiose (FC = 2.44) contributed to the alteration of the galactose metabolism. The glycine, serine, and threonine metabolism was altered via pyruvic acid (FC = 0.69), L-serine (FC = 6.38), hydroxypyruvic acid (FC = 0.4), guanidinoacetic acid (FC = 1.93), dimethylglycine (FC = 2.32) and L-cystathionine (FC = 6.14).

## 3. Discussion

The sampled turtles were considered free from abnormalities or defects upon physical examination. The biochemistry panel assessment indicated elevated uric acid, GLDH, Bile Acids Pre-prandial/Random in the impacted turtles with respect to the reference site turtles. It was noted in the clinical pathology report and upon review of the data that the elevated uric acid may be an indication of dehydration, GLDH (albeit, within normal ranges) may be an indication of increased extrahepatic and hepatobiliary disease potential, and the elevated bile acids may be a result of additional enzymes required for urea synthesis. However, the clinical pathology report indicated no concerns in terms of turtle health outside the elevated PFAS and the indications noted above. The turtles were also sampled from sites that were not known to be impacted by any other contaminants of concern.

The present study applied an integrated multi-omics approach to decipher the metabolic contribution of the host and gut microbiome of turtles exposed to elevated levels of PFAS. As previously described, the microbial community profiles showed remarkable variation between the two turtle groups (i.e., PFAS-impacted and PFAS-free reference groups). This variation, albeit with some caveats around age and food availability, suggests the elevated PFAS alters the community composition of the gut microbiota [31]. Along with changes to the gut microbiota, measurable metabolic profiles of PFAS-exposed turtles showed alterations in many faecal metabolites, compared to those of the reference turtles. The metabolite host–microbiome origin analysis performed using MetOrigin enabled these features to be attributed to their source, with many metabolites observed that are common to co-metabolism activities that are tied to both the turtle host and its inhabiting gut microbiota. Not surprisingly, some specific metabolic activities are unique to the host, and excreted into the gut microbiome, in addition to the microbiota itself that are altered. Changes in these metabolites in the PFAS-impacted turtles may be due to the impacts of PFAS on the host metabolism, which in turn impact the microbial community, or vice versa, which in turn affects the health of the host.

### 3.1. Community Composition

The analysis of the bacterial community profile identified five main components of the microbiota in turtle faecal samples, including Firmicutes, Bacteroidota, Proteobacteria, Fusobacteria, and Cyanobacteria. Among these, Firmicutes (60.52%) were the most abundant group followed by Bacteroidota (20.54%), and Proteobacteria (9.70%), which together comprised 90.75% of the total relative microbiome. The characterisation of this community composition is described in detail within our previous short communication paper, where it was highlighted that the PFAS-impacted turtles had an increased relative proportion of Firmicutes and a decreased relative percentage of Bacteroidota, compared to reference turtles [31]. Similar F/B patterns have been observed in other stressed/perturbed turtles [33,34,35]. Moreover, the relationship between the two dominant phyla, expressed as the F/B ratio, has been proposed to be a useful marker of several pathological conditions such as gut dysbiosis, obesity, aging, and inflammation [36,37].

### 3.2. Community Metabolic Function

At the metabolic level, the impacts of PFAS were clearly demonstrated through the upregulation of 56 metabolites and the downregulation of 55 metabolites in PFAS-impacted turtles compared to turtles from the reference site. A major proportion of these altered metabolites from turtles′ faecal samples originate from turtles, suggesting the impacts of PFAS on host physiology and metabolism. Indeed, the pathway analysis revealed several pathways influenced by PFAS of which 5 pathways were significantly affected (*p* < 0.05). Amino acid metabolisms were the most affected pathways, and amino acids were also the most altered metabolites, which indicates the strong effects of PFAS on amino acid metabolism. Similar to this study, the impacts of PFAS on amino acids have been reported in many studies on humans (reviewed by Gou et al. [38]) and animal models [26,28,39,40]. Since amino acids are a major source of cell energy, impaired amino acid metabolism pathways may suggest the disturbance of energy metabolism in PFAS-exposed turtles.

Another notable observation in PFAS-impacted turtles is the changes in many butyrates which leads to a significantly affected pathway of butanoate metabolism (or butyrate metabolism). Butanoate metabolism is described as the metabolic fate of short-chain fatty acids (e.g., acetate, propionate and butyrate) that are produced by bacterial fermentation of carbohydrates in the colon [41]. Hence, the change of several butyrates and butanoate metabolism itself in the faecal metabolome of PFAS-impacted turtles suggests the disturbance of PFAS on microbiota which was reflected by the high F/B ratio in these animals. The alteration of butanoate metabolism associated with PFAS concentrations has been reported in the serum of children [42] and pregnant women [43].

Purine and pyrimidine nucleotides are the building blocks of nucleic acids, components of high-energy nucleotides, and precursors for the synthesis of nucleotide cofactors [44,45,46]. Hence, purine and pyrimidine metabolisms are important for cellular energy systems and cellular homeostasis [45]. However, the disorders of purine and pyrimidine metabolism which are due to abnormalities in the biosynthesis, interconversion, and degradation of the purines and pyrimidines can result in a broad spectrum of clinical manifestations [44,47]. The association between PFAS and purine and pyrimidine metabolism has been reported in several studies in humans [42,43]. The change in purine metabolism was observed in zebrafish following the perfluorooctane sulfonate (PFOS) exposure [26]. The impact of PFAS on purine and pyrimidine metabolism was reported in turtles′ serum samples following PFAS exposure [22]. In this study, both purine and pyrimidine metabolism were significantly enriched (*p* < 0.05), and pyrimidine metabolism was identified as a significantly impacted pathway (*p* < 0.05) while purine metabolism was considered as a pathway of interest with a slight impact (*p* = 0.092).

### 3.3. Host–Gut Microbiome Metabolism

More than half of the identified metabolites are of microbiome origin. This suggests the difference in metabolomic profiling of faecal samples between PFAS-exposed turtles and references turtles may be partially explained by differences in microbiota between the two turtle groups. In addition, the correlation analysis of metabolites and microbiome showed the opposite correlation direction between bacterial phyla, especially between Firmicutes and Bacteroidota. While many metabolites are highly abundant in Firmicutes, they have low levels in Bacteroidota, and vice versa. Hence, the very high F/B ratio in PFAS-exposed turtles as discussed earlier is a strong contribution to the observed differences in the measured metabolite profiles between the two turtle groups. Similar to this study, a significant correlation of gut microbiota with differential metabolites was also observed in zebrafish [48], which contributed to an induced gut microbiota dysbiosis and hepatic metabolism disorder when exposed to PFAS (specifically, Sodium ρ-perfluorous nonenoxybenzene sulfonate (OBS)). Furthermore, these same impacts of gut microbiota on the host metabolome have been documented in human observational studies [49,50,51]. It is not surprising that gut microbes are known to produce many of the body′s chemicals, hormones, and vitamins [52] that could regulate the host homeostasis and other diverse metabolic pathways [51,53,54,55]. As such, disruption to this community, as evident by the alteration of the turtle metabolome in the present study, could be the result of the complex interaction that is observed between the host, PFAS exposure, and gut microbiota composition.

Accounting for the metabolic interaction between the host and gut microbiota of the sampled turtles, a total of 30 co-metabolic pathways were deemed to be statistically significant (*p*-Value < 0.05). Two metabolic pathways were significantly (*p*-value < 0.05) altered in the host including the steroid hormone biosynthesis and primary bile acid biosynthesis. While the levels of androsterone (FC = 0.19), 5α-pregnane-3, 20-dione (FC = 0.38), and dihydrotestosterone (FC = 0.59) seem to have decreased in PFAS-exposed turtles, the levels of 5-β-cholestane-3α,7α,12α-triol (FC = 2.42) and 25-hydroxycholesterol (FC = 1.8) were elevated. Like this study, the impact of PFAS on steroid biosynthesis, particularly testosterone, has been reported to diminish in many studies [56,57,58,59]. 5-β-cholestane-3α,7α,12α-triol and 25-hydroxycholesterol synthesise bile acids downstream. Several studies have reported similar impairment of bile acid synthesis due to PFAS exposure [60,61].

The top altered significant metabolic pathways in the microbiota involved cysteine and methionine metabolism, sulfur metabolism, toluene degradation, and tyrosine metabolism. The cysteine and methionine metabolism are altered via elevated O-acetylserine (FC = 2.98), O-succinyl-L-homoserine (FC = 1.84), and methionine sulfoxide (FC = 1.51) levels. O-acetylserine and O-succinyl-L-homoserine are also involved in sulfur metabolism. The disruptions in the cysteine and methionine metabolism and sulfur metabolism could be due to the interactions of sulfonic group of PFAS with sulfur-related biochemical processes [62]. The toluene degradation was found to be disrupted via decreased levels of 3-hydroxybenzoic acid (FC = 0.11) and (R)-2-benzylsuccinic acid (FC = 0.27). The significant enrichment of toluene degradation pathway could potentially indicate the role of toluene-degrading gut microbes on PFAS breakdown. 3-hydroxyphenylacetic acid (FC = 0.1) and tyrosol (FC = 0.15) involved in tyrosine metabolism were also reduced, which could be further linked to toluene degradation and sulfur metabolism. The disruption of tyrosine metabolism due to exposure tof OBS has been reported earlier [63]. The accumulation of tyrosine and a disturbed tyrosine metabolism is a sign of liver injury.

Key members that are more abundant in the PFAS-impacted turtle gut microbiota and tied to these metabolic changes, as indicated in Supplementary Figures S5 and S6, predominately belong to two phyla Firmicutes (especially species annotated as being *Lactococcus garvieae*, *Clostridium baratii*, *Clostridium botulinum*, *Clostridium butyricum*, *Clostridium gasigenes*, *Clostridium bornimense*, and *Lachnoclostridium phytofermentans*) and Proteobacteria (*Laribacter hongkongensis* and *Rahnella aquatilis*). While there are no published links between these members and PFAS exposure, they are known in the literature as either zoonotic pathogens or as tied to causative health implications. For example, *L. garvieae* and *L. hongkongensis* bacterium are zoonotic pathogens [64,65], *C. baratii* and *C. botulinum* are known to produce neurotoxins [66,67], *C. gasigenes* is associated with meat spoilage [68], and *R. aquatilis* has been identified as an abundant microbe in immunodeficient patients [69], but identified as a rare and beneficial microbe in snails and soils [70]. *C. butyricum* is a common gut microorganism found in the environment that has been tied to short chain fatty acid production via the carbohydrate fermentation [71]. This suggests that the changes to the microbiota, and subsequent metabolic function, are altering the community structure in a way that facilitates opportunistic pathogens and non-beneficial microbes to colonise the gut microbiome, which could further enhance the PFAS phenotypical response.

The strong correlation between microbes and metabolites in turtles’ faecal samples at both species and phylum levels may suggest that the faecal metabolome could be used to predict the microbiome composition and impact of PFAS on turtles. Zierer et al. [50] analyzed human faecal samples and found the faecal metabolome largely reflects gut microbial composition, and is strongly associated with visceral-fat mass. Hence, the authors suggested that the faecal metabolome could be used as a functional readout of the intestinal microbiota and potential mechanisms underlying microbial influence on abdominal obesity. Similarly, Zhao et al. [72] reported the correlations between disordered gut microbiota and faecal metabolites in individuals with type 2 diabetes. The strong association between faecal microbiome composition and faecal metabolite measurements have been reported in other studies in human [73,74,75,76] and animal models [48,49]. Hence, the faecal metabolome has the potential to provide useful information for the characterization of the intestinal microbiota and prediction of host phenotype.

## 4. Materials and Methods

### 4.1. Animal Ethics

Freshwater short-neck turtles (*Emydura macquarii macquarii*) were humanely killed in accordance with AEC permit SA2021/08/795 approved by the Queensland Department of Environment and Science (DES) Animal Ethics Committee (September 2021). A reciprocal review was noted at the CSIRO Wildlife, Livestock, and Laboratory Animal (CWLLA) Ethics Committee meeting (Dated October 2021).

### 4.2. Turtle Collection from a Known PFAS-Impacted Site

The sampled turtles were collected as part of an ongoing PFAS investigation led by the Qld Department of Environment and Science. As such, the location and details of the site remain confidential. The PFAS-impacted site sampled consisted of a small urban waterway known to be contaminated with PFAS. The residual Σ47PFAS concentration of the water was measured to be 32.7 ± 1.1 µg/L, as described in Beale et al. [30,31]. In doing so, PFAS was identified as the chief contaminant of concern following a risk-based monitoring approach [77]. The site was also screened for metals and other organic contaminants, which were found to be negligible [22]. Each sampled site consisted of similar fauna and flora, and a summary of the water quality data from each site is presented in Supplementary Table S7. In addition to elevated PFAS, it was observed that total dissolved salts, electrical conductivity, chloride, sodium, and potassium levels/concentrations were elevated at the impacted site with respect to the reference site. These parameters are known to fluctuate dependent on water flow and evaporation [77].

Five adult female turtles were collected from the PFAS-impacted site in September 2021. A reference site with no measurable PFAS was also selected, where an additional five adult female turtles were collected (September 2021). An aliquot of turtle serum was submitted to a veterinarian pathology laboratory (IDEXX, Brisbane, QLD, Australia) for assessment of ‘Reptile Biochemistry Comprehensive’.

### 4.3. Turtle Faecal Samples

Fresh faecal samples were collected as per Beale et al. [31]. Briefly, fresh faeces were sampled directly from the colon of euthanised turtles into sterile CyroTubes (1.2 mL, polypropylene; Merck, Truganina, Australia) and flash frozen (ethanol dry ice slurry). Samples were stored at minus 80 °C until analysed.

### 4.4. Turtle Faecal Microbiome Analysis

Bacterial 16S rRNA amplicon sequencing of the fresh turtle faecal samples was performed as per Beale et al. [31]. Briefly, DNA was extracted from a triplicate 0.25 g homogenized faecal sample per turtle using the MoBio power soil extraction kit, as per the manufacturer’s instructions. Sequencing was performed using a MinION Mk1B (Oxford Nanopore, Oxford, UK) equipped with an R9.4 flowcell (FLO-MIN106D) as per the manufacturer′s instructions. The sequencer was run using MinKNOW software (v4.3.20, Nanopore, Oxford, UK), with a data acquisition set for 72 h. Consensus sequences were used to infer organism genomic content using PICRUSt2 [78]. PICRUSt2 analysis was conducted on the acquired data (processed after) using default arguments according to the suggested workflow outlined in the software documentation. Any sequences with NSTI > 2.0 were removed from further analysis. Data were filtered with a minimum median OTU abundance threshold of 4 reads, and a variance threshold of 10% based on the inter-quartile range. Rarefaction curves reached saturation indicating a sufficient sampling depth was achieved.

### 4.5. Turtle Faecal Community Metabolome Analysis

Community metabolic profiling of freeze-dried turtle faeces was performed as per Beale et al. [31]. Central carbon metabolism (CCM) metabolites were measured on an Agilent Infinity Flex II UHPLC coupled to an Agilent 6470 Triple Quadrupole Mass Spectrometer (QqQ-MS) following Sartain [79] and Gyawali et al. [80]. Untargeted metabolites were analyzed on an Agilent Infinity Flex II UHPLC coupled to an Agilent 6546 Quadrupole Time-of-Flight Mass Spectrometer (QToF-MS) following Beale et al. [22].

Acquired CCM data were first ‘blank’ subtracted, normalised to the spiked internal standards (Succinic Acid ^13^C_2_ and L-Phenylalanine ^13^C) and sample biomass. Untargeted metabolite data were normalised to reference ions (positive mode =; negative mode =). Missing values were replaced by 1/5 of the minimum positive values of their corresponding variables. The acquired data were then normalised by the sample median, log_10_ transformed and scaled using the mean-centred value and divided by the standard deviation of each variable. Data were then subjected to univariate and multivariate statistical analysis using MetaboAnalyst 5.0 [81] and SIMCA 17.01 (Sartorius Stedim Data Analytics AB, Goettingen, Germany).

### 4.6. Statistical Analysis and Host–Microbiome Data Analysis

Turtle host–microbiome analysis was performed using MetOrigin, an online tool for the integrative analysis of the gut microbiome and discriminating between host, microbiome, and co-metabolism activities associated with microbial metabolites. The processed bacterial 16S rRNA amplicon data frame and metabolomics data frame were filtered based on low RSD threshold (30%) and normalised based on bacterial relative abundance (percentage) and log-transformed metabolites. As the turtle species sampled herein does not have a metabolic pathway curated in the KEGG database (which MetOrigin queries), a nearest match was utilised (*Chrysmeys picta*; A western painted turtle) based on the order classification of Testudines and the habitat where it is found (slow moving freshwater systems of similar climates of the northern hemisphere). Known host and microbiome metabolite and metabolic pathways were then matched to KEGG. A hypergeometric test was used to calculate *p*-values. Metabolic pathways with a log_2_ *p*-values greater than 1 are considered statistically significant and taken forward for correlation analysis (using a spearman correlation method, *p*-Value < 0.05).

Enrichment of the metabolite data (MetaboAnalyst) was then used for microbiota/co-metabolism-associated metabolic pathway selection (selecting enriched pathways with a *p*-value threshold of <0.1), which explored microbiome–metabolism linkages. A series of host, microbiome, and co-metabolism networks were then established using a fold change threshold greater than 1.2 for identified microbes and their positive and negative metabolite correlations.

## 5. Conclusions

In the present study, the integration of faecal metabolomics data with gut microbiome composition information using the MetOrigin tool enabled the complex interaction of host and gut microbiome to be investigated when exposed to elevated PFAS levels in the environment. The effects of PFAS on the turtle gut microbiome were observed to influence the microbial community structure and the expressed host and microbial metabolome measured in the sampled faeces. However, further work is needed to categorically link these host–microbiome signatures to PFAS exposure and account for any biochemical or gut microbiota variance that may arise from other physical–chemical parameters the turtles are exposed to in the environments sampled.

Our data demonstrate a variation in microbial composition between PFAS-exposed turtles and reference individuals that may contribute to the measured metabolic differences in microbiome and host faecal metabolome between the two turtle groups studied. Moreover, the F/B ratio between the reference turtles and PFAS-impacted turtles supports other perturbation observational studies and has the potential to be used as a biomarker of overall health and wellbeing in turtles. The microbial members responsible for driving key metabolic changes were from the phyla Firmicutes and Proteobacteria. Several pathways associated with the carbohydrates, energy, amino acids, nucleotide, co-factors, vitamins, terpenoids, and polyketides were disrupted in both the turtle (host) and the gut microbiota. Due to its role as an endocrine disruptor, PFAS exposure significantly alters the steroid hormone biosynthesis in the turtle. Primary bile acid bio-synthesis was significantly altered in the turtle gut due to PFAS exposure. The PFAS exposure perturbed the cysteine and methionine metabolism, sulfur metabolism, toluene degradation, and tyrosine metabolism in the gut microbiota of the turtle.

However, how PFAS enhances the Firmicutes phylum in the turtle gut is currently unknown and requires further research to explore these links that also accounts for diet and turtle age. It is envisaged from this study that metabolomics tied to microbiome sequencing can be used as a tool that identifies correlates of overall health and wellbeing that could be used as an intermediate phenotype to assess the microbial effects on the host and the gut microbiome function.

## Data Availability

The data presented in this study are available on request from the corresponding author. The data are not publicly available due to the ongoing PFAS investigation at the sampled sites.

## Supplementary Materials

The following supporting information can be downloaded at: https://www.mdpi.com/article/10.3390/metabo12080747/s1, Photos: example of an impacted and reference site turtle that was sampled; Table S1: Sampled freshwater turtle serum biochemistry data; Table S2: Summary of the significant metabolites identified by the volcano plot analyses; Table S3: Summary of metabolite enrichment analysis data that support Figure 2; Table S4: Summary of metabolite pathway data that support Figure 2; Table S5: Relative phylum abundance in sampled freshwater turtle faeces per impacted and reference site; Table S6: Metabolite assignments per source and the chemical cluster ontologies as defined by ChemRICH; Table S7: Summary of identified pathways from the MetOrigin host–gut microbiome function analysis; Table S8: Summary of water quality data from the impacted and reference sites; Figure S1: MinION 16S processed data rarefaction curves; Figure S2: identified pathways from the host-microbiome function analysis; Figure S3: Microbiome community (at the species level) and expressed metabolite correlation analysis; Figure S4: Host metabolic contribution and significantly expressed metabolites; Figure S5: Microbiome species specific metabolic contributions and significantly expressed metabolites; Figure S6: Co-metabolism contributions from the microbiome species-specific entities and the host, and the significantly expressed metabolites.

## Abbreviations

Per- and polyfluoroalkyl substances (PFAS); perfluorooctane sulfonate (PFOS); Liquid chromatography-mass spectrometry (LC–MS); central carbon metabolism (CCM); principal component analysis (PCA); fold change (FC); operational taxonomic units (OUT); metabolite identification and abbreviations are listed in Supplementary Table S6.
